# Cognitive factors and processes in models of insomnia: A systematic review

**DOI:** 10.1111/jsr.13923

**Published:** 2023-06-26

**Authors:** Nicole K. Y. Tang, Bruno Saconi, Markus Jansson‐Fröjmark, Jason C. Ong, Colleen E. Carney

**Affiliations:** ^1^ Department of Psychology University of Warwick Coventry UK; ^2^ Department of Population Health Sciences, Geisinger Danville Pennsylvania USA; ^3^ Centre for Psychiatry Research, Department of Clinical Neuroscience, Karolinska Institutet, & Stockholm Health Care Services, Region Stockholm Stockholm Sweden; ^4^ Nox Health, Inc Alpharetta Georgia USA; ^5^ Department of Psychology Toronto Metropolitan University Toronto Ontario Canada

**Keywords:** CBT‐I, cognition, cognitive behaviour therapy, insomnia, theory, thoughts

## Abstract

Cognition is central to the experience of insomnia. Although unhelpful thoughts about and around insomnia are a primary treatment target of cognitive behaviour therapy for insomnia, cognitive constructs are termed and conceptualised differently in different theories of insomnia proposed over the past decades. In search of consensus in thinking, the current systematic review identified cognitive factors and processes featured in theoretical models of insomnia and mapped any commonality between models. We systematically searched PsycINFO and PubMed for published theoretical articles on the development, maintenance and remission of insomnia, from inception of databases to February, 2023. A total of 2458 records were identified for title and abstract screening. Of these, 34 were selected for full‐text assessment and 12 included for analysis and data synthesis following the Preferred Reporting Items for Systematic Reviews and Meta‐Analyses (PRISMA) guidelines. We identified nine distinguishable models of insomnia published between 1982 and 2023 and extracted 20 cognitive factors and processes featured in these models; 39 if sub‐factors were counted. After assigning similarity ratings, we observed a high degree of overlap between constructs despite apparent differences in terminologies and measurement methods. As a result, we highlight shifts in thinking around cognitions associated with insomnia and discuss future directions.

## 1 INTRODUCTION

Research advances have left little doubt that the causes of insomnia are multi‐factorial in nature, from genetics and physiology to brain mechanisms and psychology (Riemann et al., 2022; Van Someren, 2021). Whilst no one single approach fully explains the development and maintenance of insomnia, understanding of the psychological factors underpinning the perpetuation of insomnia has fruitfully led to the development of effective non‐pharmacological treatments for insomnia. In particular Cognitive Behaviour Therapy for Insomnia (CBT‐I), which has garnered robust evidence of efficacy and effectiveness and is now recommended as the first‐line treatment by multiple learned societies across continents (National Institute for Health and Care Excellence, 2022; Qaseem et al., 2016; Ree et al., 2017; Riemann et al., 2017; Wilson et al., 2019). Despite these successes, CBT‐I is not a one‐size‐fits‐all panacea. Around 40% of patients who received CBT‐I did not show “therapeutic response” (defined as Insomnia Severity Index, ISI, change score >7) whilst 60% did not achieve “clinical remission” (defined as ISI score <8) at post‐treatment (Morin et al., 2009; Morin & Benca, 2012). And whilst CBT‐I's positive treatment effect is generally well‐maintained, only 50–60% of patients achieve remission 6–12 months after treatment (Muench et al., 2022).

In considering future directions of CBT‐I, multiple opinion leaders recommend exploring ways to maximise treatment outcomes, identifying pathways that mediate treatment effect, and addressing factors associated with non‐response (Harvey & Tang, 2003; Kyle et al., 2013; Manber et al., 2015; Muench et al., 2022; Vitiello et al., 2013b, 2013a). It seems reasonable to think that continual advances of CBT‐I would in part rely on better understanding of the cognitions and behaviours underpinning persistent insomnia because CBT‐I involves methods and interventions that intend to change these.

In CBT, behaviour and cognitions are intricately linked. The role of cognitions has come to the forefront riding on the so called “cognitive revolution” of psychological research which saw the addition of a cognitive mediator in the direct stimulus–response model of behaviour (Gelder, 1997; Rachman, 1997). Cognitions refers to mental events such as thoughts and beliefs. Though not observable by others, cognitions can be consciously accessed and measured via self‐report. The cognitive perspective has been applied to conceptualise a range of emotional disorders (e.g., depression, specific phobia, panic disorder, social phobia, posttraumatic stress disorder, obsessive compulsive disorder, health anxiety, eating disorder, generalised anxiety disorder) and subsequently to a range of physical health conditions that are compatible with the biopsychosocial framework, including insomnia. Across these models, the general principles remain the same, in that our thoughts and interpretations of a certain situation could influence emotions and physiology, as well as what we do in response. The specific cognitive constructs of interest, however, differ between fields of applications in form (e.g., attention, perception, appraisal, information processing, memory, imagery) and level (e.g., core beliefs, intermediate beliefs and attitudes, automatic thoughts, metacognitions).

Most people with insomnia identify “cognitive arousal” over “somatic arousal” as the main cause of their sleep problems (Lichstein & Rosenthal, 1980). This is consistent with clinical observations over the years of a “racing mind” as the crux of the insomnia experience (Borkovec, 1982; Espie, 2007; Geer & Katkin, 1966; Harvey, 2001; Spielman et al., 1987). However, the definition and operationalisation of such “racing mind” and cognitive arousal in general vary between theoretical models of insomnia. Their roles in the initiation and maintenance of insomnia also differ, with some investigators on the one hand suggesting cognitive arousal is a mere epiphenomenon of nighttime wakefulness (Freedman & Sattler, 1982), whereas some other investigators proposing that they are key drivers or mediators of the vicious cycle of insomnia (Harvey, 2002; Morin et al., 2003).

Since multiple cognitive models of insomnia have been proposed over the years, the aims of this systematic review are therefore to: (1) identify, describe, and review published models of insomnia that have featured cognition as a factor contributing to the development, maintenance and/or remission of insomnia; (2) map common cognitive factors shared between models; and (3) to highlight shifts in thinking in cognitive conceptualisations of insomnia over time and consider future research and clinical directions.

## 2 METHODS

### 2.1 Searches

Searches were performed in PubMed and PsycInfo, the two most relevant electronic databases that cover publications in medicine and psychology in general, using tailored search terms developed by two of the authors (NT, BS) jointly with two information specialists (JG, SJ) to reflect the pre‐specified inclusion and exclusion criteria (see Appendix A on online supporting information for detailed search strategy). Each of these databases was searched from inception to October 2022. The searches were updated in February 2023. The protocol for this systematic review was not prospectively registered, though we followed the PRISMA guideline (Page et al., 2021) for reporting (see Appendix B for a checklist).

### 2.2 Eligibility criteria

In terms of inclusion criteria, articles were included if they are (1) theoretic articles, defined as manuscripts examining existing evidence with the explicit goal of generating new theoretical frameworks; (2) on chronic or persistent insomnia; (3) providing an original perspective on the role of psychological factors in the development, maintenance and/or remission of insomnia disorder; (4) in humans; (5) with at least one of the factors featured in the theory/model is concerned with cognitions, which is broadly defined as mental events such as attention, thoughts/beliefs, perception, appraisal, information processing, and memory etc.

Articles were excluded if their focus was on (1) acute insomnia or sleep disorders in general, (2) comorbid insomnia (e.g., insomnia disorder comorbid with depression, anxiety disorder, posttraumatic stress disorder, dementia, head‐injury, cancer, HIV/AIDS, chronic obstructive pulmonary disease, pain), (3) explaining the link of insomnia with another medical or psychiatric condition, another sleep disorder or substance (mis)use, (4) the effect of sleep disturbances on cognitive decline, (5) insomnia management strategies and their effectiveness, rather than the nature of cognitive processes that manifest or mitigate insomnia, (6) insomnia in specific groups (e.g., individuals with a history of abuse, pregnant women, older adults in care home, prisoners), (7) infants who are yet to develop a stable circadian rhythm and sleep patterns. Articles were also excluded if they (8) primarily summarised – qualitatively or quantitatively – existing literature without proposing new theories or perspectives, (9) primarily discussed the role of emotion/affect/mood in insomnia, (10) their full text was not in English.

### 2.3 Search strategies and data extraction

Hits returned were downloaded and imported to COVIDENCE (https://www.covidence.org/), an auditable online platform for systematic review management. COVIDENCE was used by the review team to manage search results, remove duplicates, perform the screening and data extraction, and store study information. Reference lists of papers identified for inclusion of full‐text screening were hand‐searched to identify any relevant literature missed.

After removing duplicates of hits between databases, two of the authors (NT, BS) performed the title and abstract screen on the unique returns independently. Any discrepancies were discussed and resolved between the reviewers, erring on the side of caution (i.e., passing an article onto full‐text screen if there was insufficient information to support inclusion/exclusion).

Full texts of the papers considered potentially eligible were obtained. The authors then independently assessed each paper against the inclusion and exclusion criteria. Studies that did not meet all inclusion and exclusion criteria were excluded, with reasons provided. Any discrepancies between the two reviewers at the full‐text screening stage were resolved, in consultation with the wider review team.

### 2.4 Synthesis approach

Given the theoretical nature of the articles included, data extraction was carried out by two of the authors (NT, BS) reiteratively using a pro‐forma with open text description. The authors are both clinical researchers interested in psychological models of insomnia, but have different training backgrounds (Psychology; Nursing), theoretical affiliations (CBT; Biopsychosocial), and familiarity (Primary research areas: CBT‐I, Insomnia and Chronic pain; Sleep, OSA and Chronic Pain). Their interpretation of findings was cross‐checked with all other authors in the final stage of manuscript preparation.

Study characteristics (e.g., year and country of publication, publication type) and model characteristics such as cognitive factor(s), conceptual definition(s), and model figures (if available) were extracted from each publication. The data extraction was performed with the aims to provide a summary of the key idea(s) and key cognitive factor(s) of each identified theory/model. Common and unique feature(s) across the identified theories/models were also highlighted in the results. Where more than one article was published on iterations of the same theory, these articles were considered and described in conjunction to enhance coherence and to reflect the progression/refinement of ideas over time.

Cognitive factors within models were identified and conceptual similarity of constructs across models was graded as “high” or “low”, for visualisation. Highly similar constructs had shared nomenclature, conceptualisation, were featured as core component(s) within model, and/or were cross‐referenced with previous models. Low similarity was indicated if cognitive factors had little overlap and if there was no or minimal cross‐referencing of concepts between them in their respective models.

## 3 RESULTS

### 3.1 Study selection

**FIGURE 1:**
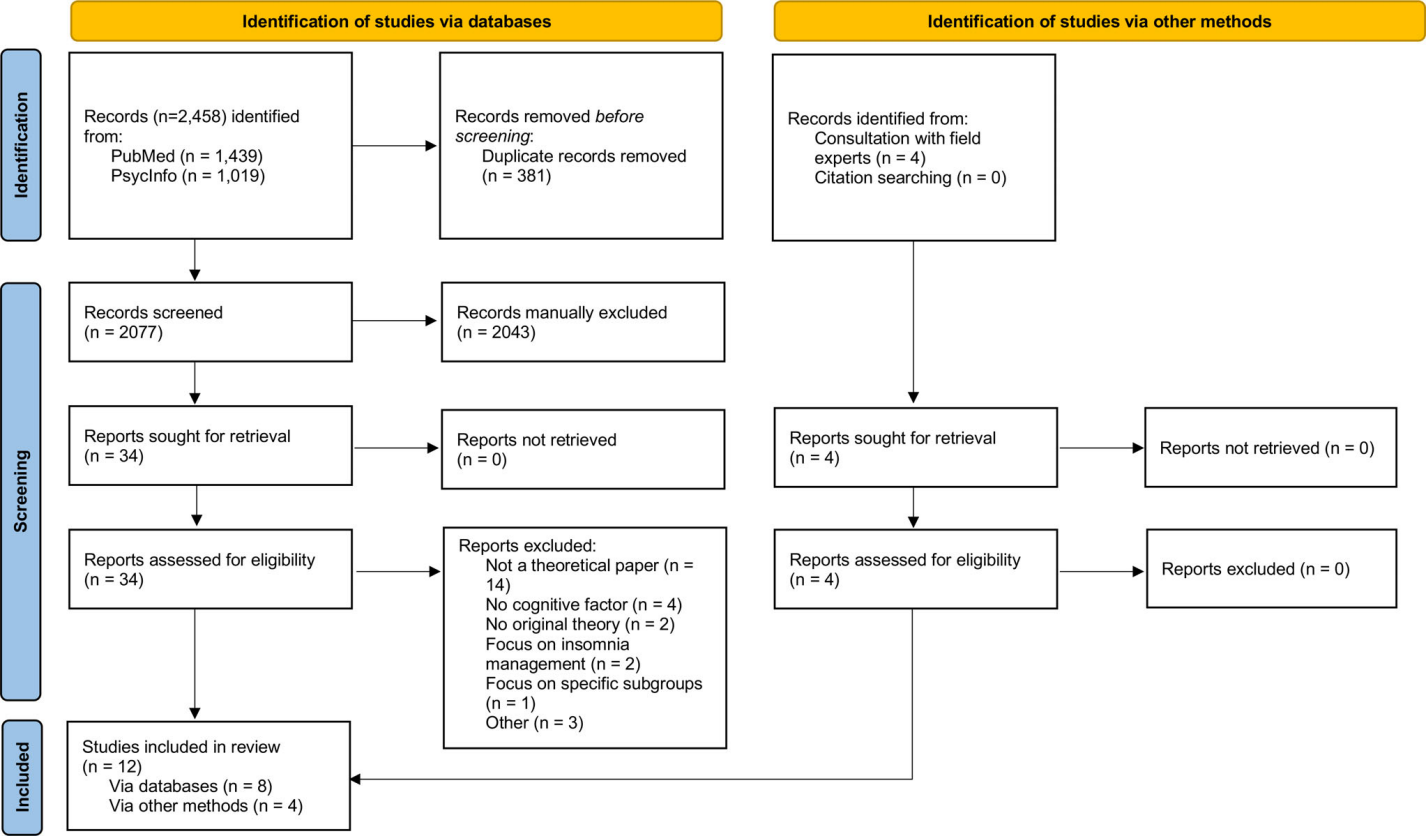
PRISMA flow diagram [Color figure can be viewed at wileyonlinelibrary.com]

A total of 2458 records were identified through electronic data search. Upon duplicates removal, 2077 studies remained. After title and abstract screening, 34 studies were selected for full‐text assessment. Additionally, one book chapter (Morin, 1993) and three articles (Borkovec, 1982; Espie, 2023; Fichten et al., 2001) that had not been captured by primary electronic searches were manually added to full‐text review. The most common reasons for exclusion were not being a theoretical paper (*n* = 14) and absence of cognitive factors on proposed theoretical model (*n* = 4). In all, 12 publications were selected for analysis and data synthesis (Figure 1).

### 3.2 Study characteristics

**FIGURE 2:**
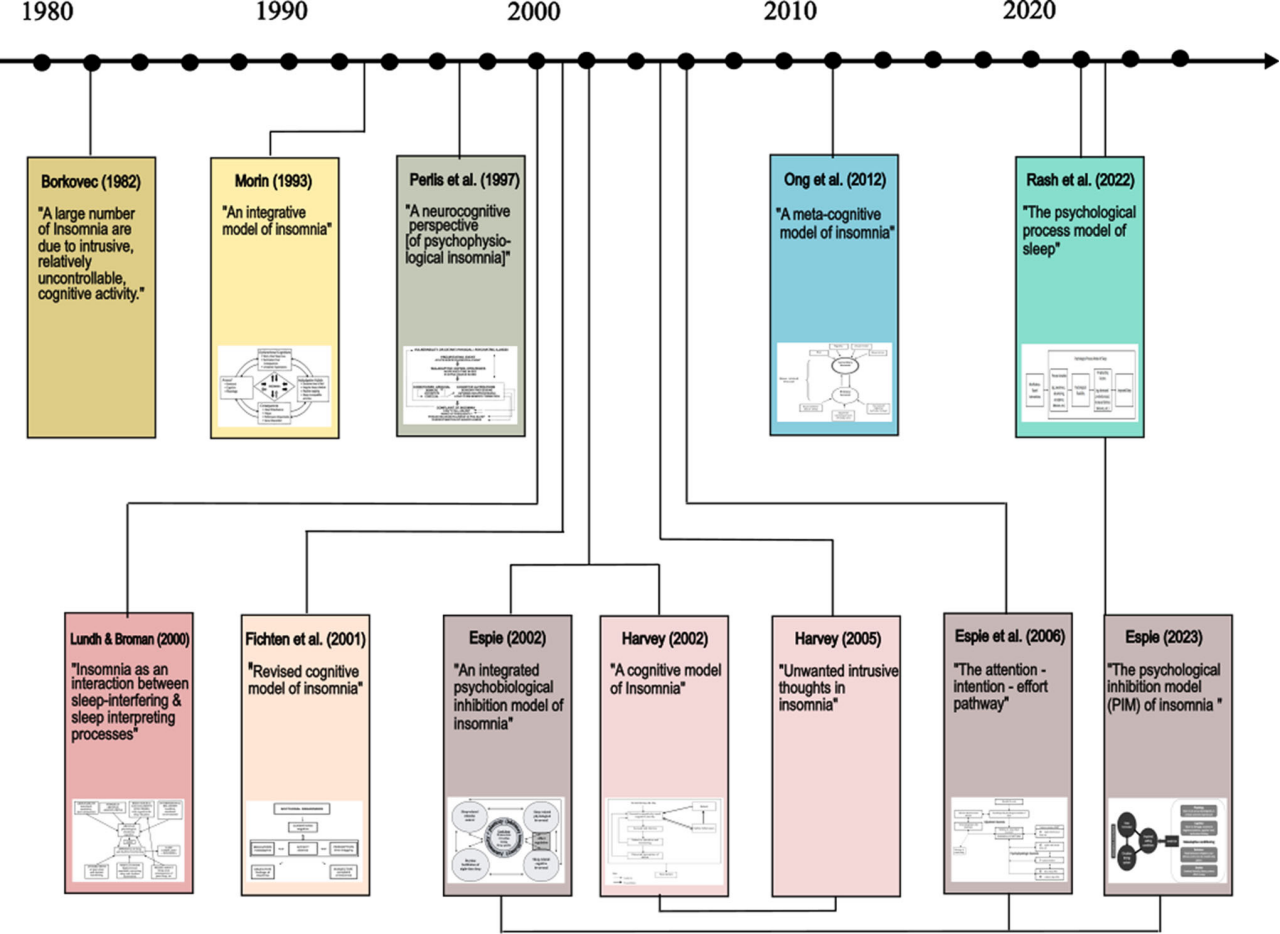
A timeline of the identified theories/models of insomnia featuring cognitions [Color figure can be viewed at wileyonlinelibrary.com]

Ten articles (Borkovec, 1982; Espie, 2002, 2023; Espie et al., 2006; Fichten et al., 2001; Harvey, 2002; Lundh & Broman, 2000; Ong et al., 2012; Perlis et al., 1997; Rash et al., 2019) and two book chapters (Harvey, 2005; Morin, 1993) describing nine distinguishable models of insomnia were included (Figure 2). These were published between 1982 and 2023, with the majority published between 1992 and 2006 (66.7%, 8/12). Most studies were led by authors in the USA (41.7%) (Borkovec, 1982; Harvey, 2002, 2005; Ong et al., 2012; Perlis et al., 1997), followed by the UK (25%) (Espie, 2002, 2023; Espie et al., 2006), Canada (25%) (Fichten et al., 2001; Rash et al., 2019; Morin, 1993) and Sweden (8.3%) (Lundh & Broman, 2000). Articles were published in psychology (50%) or sleep‐related scientific journals.

Of the nine models identified, two specifically sought to explain psychophysiological insomnia only (Espie et al., 2006; Perlis et al., 1997), one psychophysiological and subjective insomnia (Borkovec, 1982), three “primary”/chronic insomnia (Harvey, 2002; Ong et al., 2012; Rash et al., 2019). Insomnia type was not specified in the remaining models. The identified models highlighted cognitive factors involved with the development (Borkovec, 1982; Espie, 2002, 2023; Espie et al., 2006; Lundh & Broman, 2000; Morin, 1993; Ong et al., 2012; Perlis et al., 1997; Rash et al., 2019), maintenance (Borkovec, 1982; Espie, 2002, 2023; Espie et al., 2006; Fichten et al., 2001; Harvey, 2002; Harvey, 2005; Morin, 1993; Ong et al., 2012; Rash et al., 2019), and remission (Borkovec, 1982; Espie, 2002, 2023; Espie et al., 2006; Harvey, 2002, 2005; Morin, 1993; Ong et al., 2012; Rash et al., 2019) of insomnia. For two of the models (Espie, 2002; Harvey, 2002), we identified additional theoretical papers elaborating on aspects of the theories.

All models were conceptualised for adults and the extent to which the models apply to children and adolescent populations was not specified.

### 3.3 Models’ description

**FIGURE 3:**
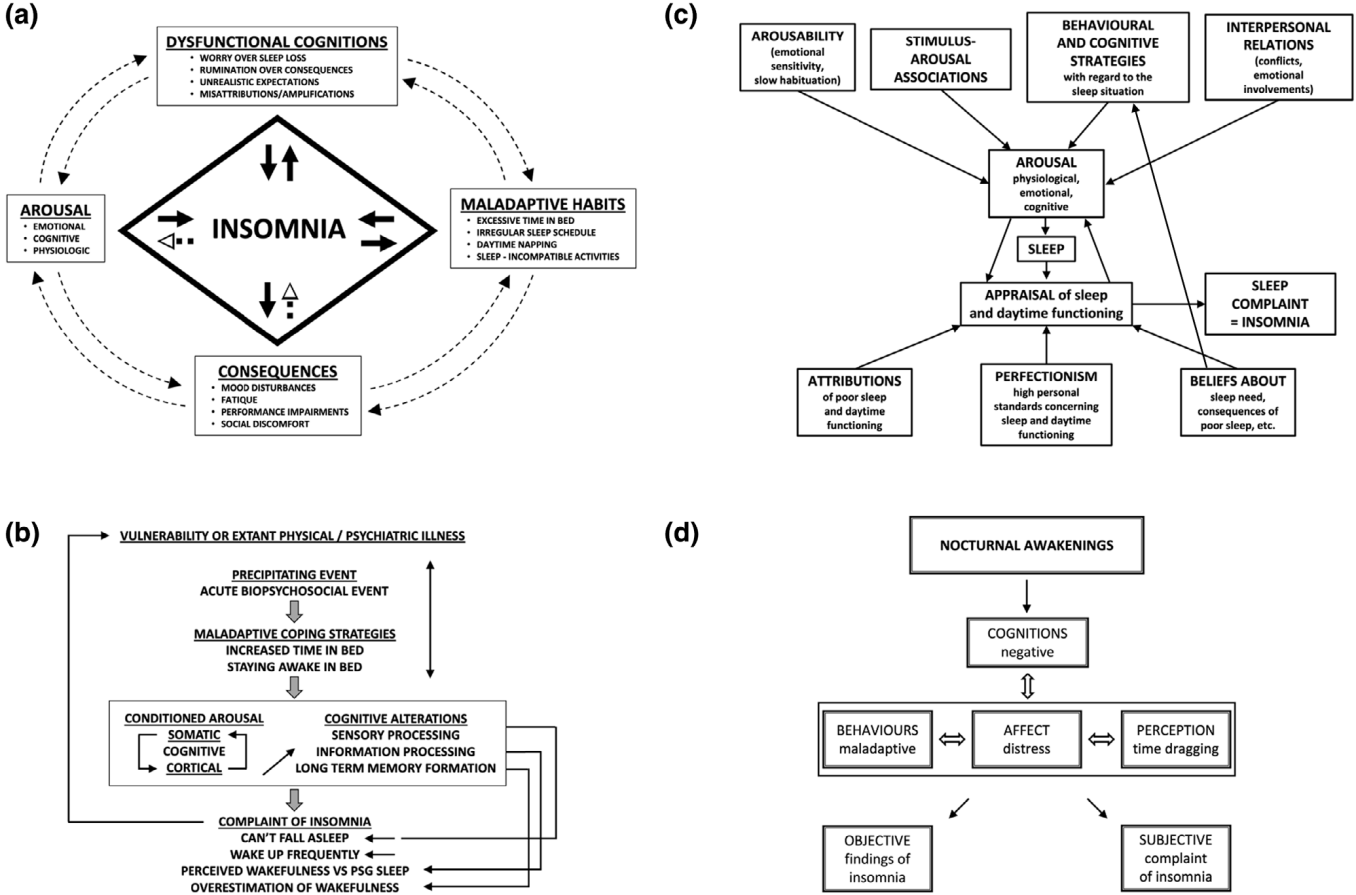
Schematic representations of models originally published before 2002. (a) Morin (1993), (b) Perlis (1997), (c) Lundh & Broman (2000), (d) Fichten et al. (2001). Reproduced with permission.

**FIGURE 4:**
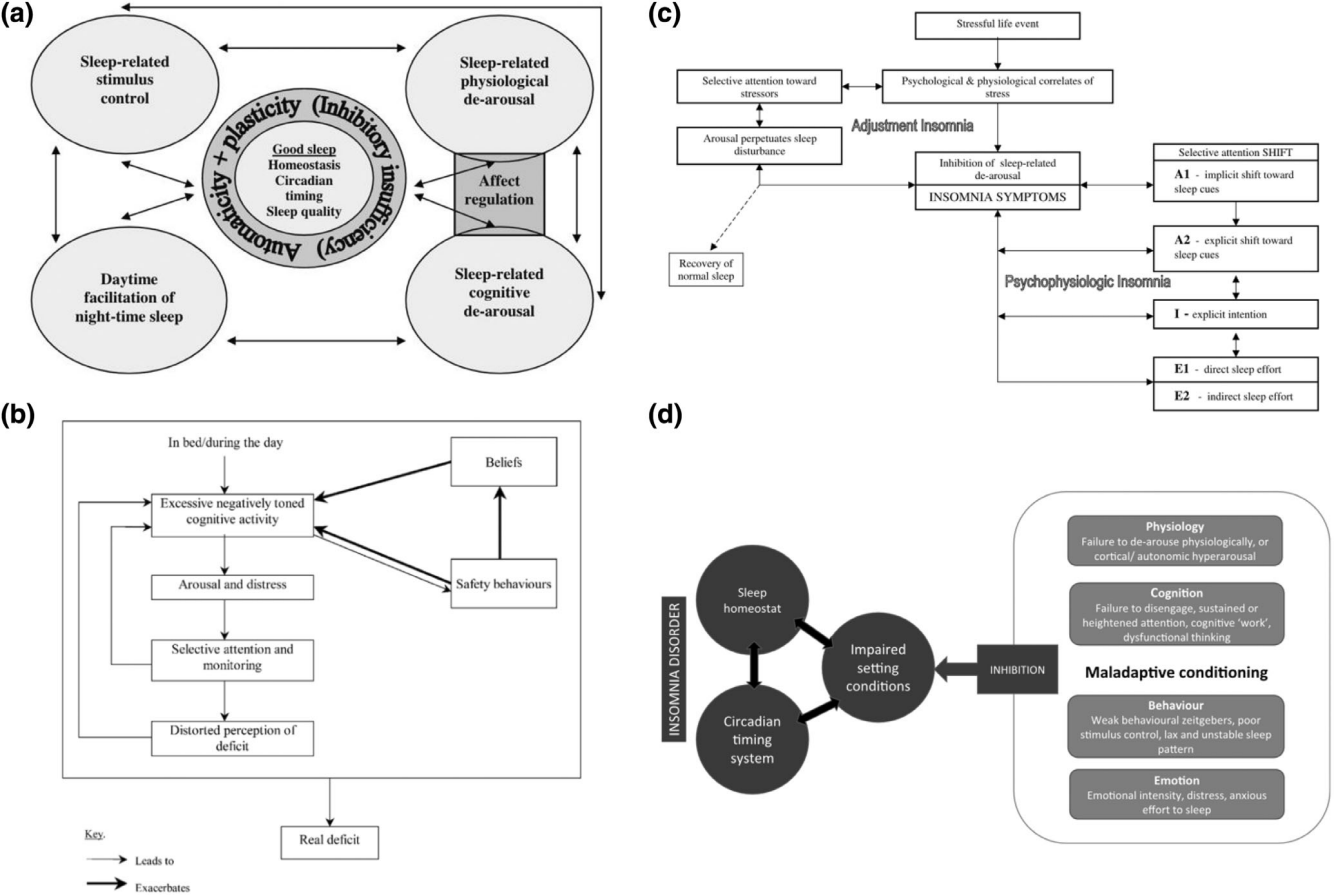
Schematic representations of models originally published between 2002 and 2011. (a) Espie (2002), (b) Harvey (2002), (c) Espie et al. (2006), (d) Espie (2023). Model d was originally published in Espie (2002) and reproduced/updated in Espie (2023). Reproduced with permission.

**FIGURE 5:**
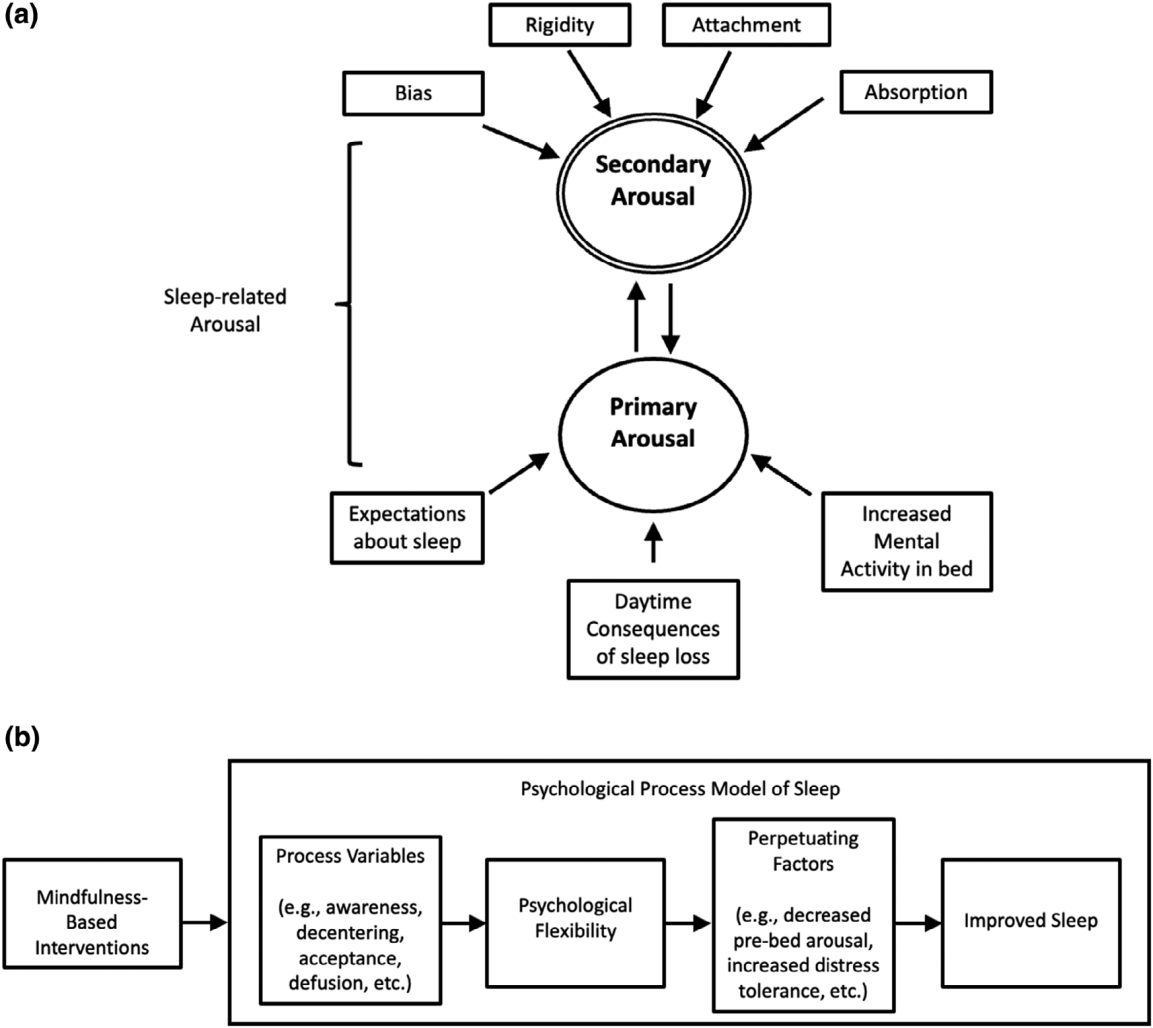
Schematic representations of models originally published after 2011. (a) Ong et al. (2012), (b) Rash et al. (2019). Reproduced with permission.

Below we provide a summary of the nine distinguishable models we identified, with particular emphasis placed on the cognitive factors mentioned in these models. Models are presented chronologically (Figures 3, 4, 5).

#### 3.3.1 Borkovec (1982)

In this early psychological theory, worry was hypothesised as the central cognitive factor that underlies sleep‐onset insomnia. Worry in Borkovec's (1982) theory referred to intrusive, uncontrollable thoughts, and images that are attention‐grabbing in nature. These cognitions are not necessarily negative in tone, but engagement in worry can result in arousal that may interfere with the body's engagement with sleep mechanisms. Worry can also lead to a wake‐like phenomenon during lighter stages of sleep, that has been linked to distorted time perception. Borkovec (1982) suggested that worry may be a product of a “obsessively worrisome cognitive style” characteristic of many people with insomnia (p. 892). The focus, frequency and intensity of worry can vary from day to day, as an interaction with the environment. Importantly, worry was thought to be a cognitive factor that can be accentuated by a bedroom environment, which is typically a sensory deprived environment making it easier for cognitive intrusion to be noticed, and can be elicited or reinforced by association (i.e., conditioning). This helped to explain how insomnia was maintained and why therapeutic effects were found for treatment strategies (e.g., relaxation, stimulus control, paradoxical intention) that act to terminate or direct attention away from sleep‐incompatible intrusive cognitions. Borkovec (1982), however, acknowledged that insomnia is a heterogeneous concept and restricted the application of his proposal to “sleep onset insomnia of the psychophysiological and subjective subtypes” (p. 891).

#### 3.3.2 Morin (1993)

Morin took the view that chronic insomnia does not develop dramatically overnight, but is a gradual evolution from situational insomnia that fails to improve even when the precipitating stressor is removed. In his integrative model of insomnia, hyperarousal was posited as the common pathway to insomnia. Once the arousal level, be it cognitive, emotional or physiological, is raised above a critical threshold, the body's natural sequence to sleep onset would be interrupted. Morin (1993) suggested that these arousals can be conditioned by both night time and daytime events and that learned behavioural and cognitive responses play a crucial role in perpetuating insomnia, with appraisal of the initial sleep difficulty as a determining factor of chronic insomnia. Such appraisal could take the form of worry/rumination/amplification over sleep loss and its daytime consequences, unrealistic expectations about sleep, misattribution of causes of insomnia, and unhelpful beliefs about sleep‐promoting practices. These cognitive factors are collectively named as cognitive distortions in Morin's model and are best assessed and exemplified by the Dysfunctional Beliefs and Attitudes About Sleep Scale (DBAS). Alongside maladaptive sleep behaviours/habits, these dysfunctional sleep cognitions are posited to provoke emotional distress and intensify insomnia, and therefore constitutes a primary target for intervention.

#### 3.3.3 Perlis et al. (1997)

Based on the observation that people with insomnia typically present high frequency EEG activity in the Beta (*14–32 Hz*) and Gamma (*>32 Hz*) ranges at or around sleep onset, Perlis et al. (1997) proposed that this form of cortical arousal is a primary feature of chronic insomnia. Although the phenomenology of interest is situated within the neurocognitive level – in contrast to other models of insomnia with a more clinical focus, these high frequency EEG activities were thought to be an analogue of cognitive arousal, representing the presence of high‐level cognitive processes that are normally diminished and/or suppressed during normal sleep (i.e., sensory processing, information processing, and long‐term memory formation). Specifically, it was suggested that – due to the enhanced sensory processing – individuals with insomnia are more responsive to perturbing environmental stimuli and, thus have more trouble falling/staying asleep. Due to the enhanced information processing – and thus awareness of events – occurring around sleep‐onset and during sleep, people with insomnia experience greater difficulty distinguishing sleep from wakefulness and have a greater tendency to perceive lighter stages of sleep as wake. Further, due to enhanced formation of long‐term memory during the sleep initiation period, people with insomnia can draw on information not accessible by people without insomnia when making judgement about their sleep and may help to explain the average tendency in insomnia to overestimate the amount of time awake and to underestimate time asleep.

#### 3.3.4 Lundh and Broman (2000)

In their integrative model, Lundh and Broman (2000) introduced a framework that distinguishes “sleep‐interfering processes” from “sleep‐interpreting processes” to conceptualise insomnia. The former processes aligned closely with the arousal concept and referred to all kinds of physiological, behaviour, cognitive, and interpersonal vulnerability factors that may predispose, result in, or prohibit recovery from a state of hyperarousal. The latter processes were thought to be key in driving complaints of insomnia. They were concerned with attitude, beliefs, personal standards that influence a person's interpretation of their own sleep difficulties, and may interact with sleep‐interfering processes in maintaining and exacerbating insomnia. Cognitive factors highlighted in the Lundh and Broman (2000) model are not dissimilar from those featured in previous theories, such as negatively valenced cognitive activity, worry about sleeplessness and its adverse daytime consequences, unhelpful sleep expectations, and attributions of insomnia. However, the inclusion of perfectionism with regards to sleep and daytime functioning as a vulnerability factor is novel. Their explicit acknowledgment that insomnia is a “complaint” determined by the interaction of sleep‐interfering and sleep‐interpreting processes has highlighted the value of addressing people's subjective appraisal of sleep and daytime functioning.

#### 3.3.5 Fichten et al. (2001)

With a focus on sleep fragmentation experienced by many older people, Fichten et al. (2001) proposed a revised cognitive model of insomnia featuring negative cognitive activity as an important mediator of insomnia complaints. Negative cognitive activity in this model referred specifically to “negative, worrying, and anxious thoughts and self‐statements during periods of nocturnal wakefulness” (p. 666). Whilst not considered the root cause of insomnia, these negative thoughts were thought to be the “experiential component of physiologic cortical arousal” (p. 687). Negative thoughts can interfere with returning to sleep, cause negative affect, lead to maladaptive sleep‐related behaviour and distorted perception of time passage. And hence, influencing both “objective” findings of insomnia and “subjective” distress and complaint of insomnia.

#### 3.3.6 Espie (2002, 2023), and Espie et al. (2006)

In contrast to other models that emphasise sleep pathology, Espie (2002) proposes a psychobiological model of good (or normal) sleep as a starting point for understanding the development, maintenance, and treatment of insomnia. Good sleep is seen as the natural state of the human organism and under normal circumstances the homeostatic and circadian processes “default” to good sleep. The “core” of normal sleep is maintained by an involuntary, harmonious interaction between the circadian system and homeostatic sleep drive, and associated perceived sleep quality. Automaticity (“involuntary nature of the well‐adjusted schedule”) and functional plasticity (capacity to “absorb and readjust” to real life situations) are assumed protective properties of good sleep. These protective properties are maintained by four interacting subsystems: physiological de‐arousal, cognitive de‐arousal, sleep‐related stimulus control, and daytime facilitation of night‐time sleep. In the absence of strong enough inhibition that outweighs the stable sleep pattern (i.e., “inhibitory insufficiency”), normal sleep is maintained. In this context, insomnia is proposed as resulting from chronic inhibition of one or more of the component processes, or in other terms, a persistent loss of expression of normal sleep.

Building upon the psychobiological model of insomnia, Espie et al. (2006) emphasises the role of “automaticity”, along with the circadian and homeostatic systems, in the regulation of sleep among good sleepers. Via the attention‐intention‐effort (A‐I‐E) pathway, good sleepers passively or involuntarily respond to internal and external cues that “act as automated settings conditions for sleep” (p. 216). As explained by the authors, “the good sleeper approaches sleep, just as s/he walks or talks – without thinking much about it and without a consciously explicit plan” (p. 216). This automatic sleep schedule is inhibited when attention, intention, and/or effort (A‐I‐E) are put into the sleep engagement process. According to the AIE pathway, selective attention to sleep (i.e., sleep‐related attention bias) is driven by factors such as preoccupation about sleeplessness, worry following sleep “deprivation”, and the longing for sleep. Following attentional focus on sleep, explicit intentive actions are “designed to deliver sleep and to eliminate wakefulness” (p. 229). Such intentions are counterproductive and further inhibit the automaticity of the normal sleep observed in good sleepers. The third factor, “effort”, whether cognitive (e.g., suppression of mental stimulation) and/or behavioural (e.g., going to bed earlier) is conceptualised as a development of intention. Sleep effort is often manifested by actively attempting to sleep and increase sleep opportunity. It is worth noting that the A‐I‐E pathway is described in terms of overlapping stages as opposed to discrete components.

After over 20 years of his original publication, Espie (2023) revisited the psychobiological inhibition model (PIM) and its related A‐I‐E pathway. Espie re‐presents the four subsystems associated with sleep facilitation as physiological de‐arousal, cognitive de‐arousal, behavioural consolidation, and emotional neutrality. These subsystems are proposed in a conditioning paradigm, which may be *adaptive* and promote good sleep or *maladaptive* and inhibit good sleep resulting in insomnia disorder. Great emphasis is placed on PIM as a generic and unifying framework that is able to accommodate insights from other insomnia models. PIM is also presented as a personalisable framework as it allows for varied aetiology and perpetuating circumstances and is thus suitable to guide the selection of specific cognitive and behavioural therapeutics for treating insomnia in a personalised manner.

#### 3.3.7 Harvey (2002, 2005)

Building upon the theoretical and empirical work underpinning cognitive models of anxiety disorders, Harvey (2002) proposed a cognitive model to delineate the cognitive processes contributing to the maintenance of insomnia. The model draws on earlier psychological conceptualisations of insomnia (Borkovec, 1982; Espie, 2002; Lundh & Broman, 2000; Morin, 1993; Perlis et al., 1997) but highlights the cognitive processes that differentiate people who experience occasional insomnia from those whose difficulty sleeping turns into a chronic problem. The model describes a cascade of cognitive processes experienced by people with insomnia in bed during the night, as well as during the day. In both scenarios, negatively toned cognitive activity about not getting enough sleep and the impact of sleep problems was thought to be a trigger of autonomic arousal and emotional distress, activating a state of anxiety. In such an anxious state, attentional biases towards threat (e.g., sleep incompatible events and cues of poor health or daytime performance) and automatic monitoring of these threats within the body and in the surroundings are expected to take place. It was also suggested that the preferential allocation of attentional resources to detect sleep‐related threats increases the likelihood of detecting “evidence” of not sleeping and/or not functioning optimally due to insomnia, reinforcing the perception of sleep or daytime performance deficits and further motivating worries about sleeplessness and threat‐monitoring. Additionally, the vicious cycle can be aggravated by unhelpful beliefs about sleep (such as those described by Morin) and safety‐seeking behaviours adopted by people with insomnia may inadvertently serve to prevent disconfirmation of those unhelpful beliefs (e.g., thought suppression, imagery control). With the vicious cycle not being self‐correcting, people with insomnia can become progressively more absorbed and preoccupied by their sleep problems, night and day.

The intrusive nature of the negatively toned cognitive activity was further elaborated in a subsequent chapter by Harvey (2005), who then also explained how sleep beliefs, metacognition, thought suppression and misperception of sleep can impact on the persistence of these intrusive thoughts, i.e., whether they would be selected for further attention in the form of worry or rumination. Of the four cognitive processes highlighted, metacognition was the only factor not featured in the 2002 model. It is an umbrella term referring to thoughts and beliefs that people hold about their thoughts (Wells & Capobianco, 2020). One metacognition considered to be particularly relevant to the persistence of insomnia was positive beliefs about worry. It was suggested that positive beliefs about worry (e.g., “helps to sort things out in my mind”) would promote worrying in bed, which would in turn feedback to “excessive negatively toned cognitive activity” and kick start the whole cascade of cognitive activity maintaining insomnia as described in Harvey (2002).

#### 3.3.8 Ong et al. (2012)

As a refinement to existing concepts of cognitive arousal, Ong et al. (2012) proposed to incorporate metacognition about insomnia (i.e., thinking about thinking about insomnia) alongside unhelpful mental activities such as worry, dysfunctional beliefs and attitudes about sleep and daytime consequences that have been identified in previous cognitive models of insomnia (Espie, 2002; Fichten et al., 2001; Harvey, 2002, 2005; Morin, 1993). In this two‐level model, metacognitions about insomnia are thought to be a source of secondary cognitive arousal, which serves to amplify the negative tone of thoughts about insomnia at the primary level, further increasing attentional, perceptual, and emotional biases that perpetuate insomnia. Whilst the role of these metacognitions of insomnia are not dissimilar to the sleep‐interpreting processes described in Lundh and Broman (2000), in characterising the cognitive factors that give rise to secondary arousal Ong et al. (2012) drew on the theoretical framework underpinning the third wave therapies in which one's relationship with thoughts is considered to be more important than the content and structure of thoughts (Hayes et al., 2004). Hence, the cognitive elements thought to fuel secondary arousal were rigidity in sleep‐related behaviour and beliefs, excessive attention towards sleep‐seeking or sleep aversive thoughts and behaviour, emotional attachment to sleep, over absorption in solving the sleep problem. The metacognitive model of insomnia further hypothesised that mindfulness and acceptance‐based approaches to the treatment of insomnias that target secondary arousal by promoting psychological flexibility, balance, equanimity and commitment to values would have beneficial downstream effect on the primary arousal that interferes with sleep, thereby reducing symptoms of insomnia. This approach is in contrast to traditional approaches such as CBT‐I, which targets primary arousal with hypothesised “upstream” effects on secondary arousal.

#### 3.3.9 Rash et al. (2019)

In an attempt to explain the therapeutic effect of mindfulness‐based therapies (MBTs) for insomnia, Rash et al. (2019) proposed to adopt an overarching psychological process model of sleep. The central hypothesis is that MBTs may improve sleep by promoting psychological flexibility, which refers to the ability to stay in contact with difficult thoughts, feelings and bodily sensations, and to accept and navigate through situations according to personal values (Hayes et al., 2006; Hayes & Plumb, 2007). Accordingly, the process variables thought to be relevant to insomnia include those specified in the generic ACT model (Hayes et al., 2012): (1) acceptance, (2) cognitive defusion, (3) contact with the present, (4) self‐as‐observer, (5) values, and (6) committed action. These processes are non‐specific to insomnia, but their cultivation via MBTs is thought to help develop psychological flexibility, with possible beneficial effects on sleep‐interfering, sleep interpreting and meta‐cognitive processes (as outlined in Harvey, 2002, 2005; Lundh & Broman, 2000; Morin & Espie, 2003; Ong et al., 2012; Perlis et al., 1997), facilitating natural sleep‐related dearousal, as described in Espie (2002).

### 3.4 Trends and observations

In Table 1, we listed all key cognitive components extracted from each identified model. A total of 20 cognitive factors were extracted; 39 if sub‐factors were counted. We made an attempt to highlight similarities between constructs based on definitions provided. This was an interpretative process through which we derived several observations detailed below: Despite the distinctive focus and terminologies used in each of the nine models, there is a surprising amount of similarities and correspondences between conceptualisations. This is likely due to the fact that all models are built upon the same bedrock of empirical research into cognitive phenomena associated with insomnia. Similar clinical phenomena and puzzles are noted across models (e.g., elevated levels of pre‐sleep cognitive arousal; perceived deficits and complaints of sleep and daytime performance) but proposed explanations of how these come into being or are being intensified and maintained are different. For example, worry – or essentially enhanced negatively toned cognitive arousal – is the most shared cognitive factor across models. It is considered as a response to insomnia resulting in hyperarousal in the Borkovec (1982), Morin (1993), Perlis et al. (1997), Fichten et al. (2001), Lundh and Broman (2000), Harvey (2002) and Ong et al. (2012) models. However, in the Espie et al. (2006) and Rash (2019) models, worry is considered as an “activating agent” that hijacks attentional processes associated with natural dearousal, interfering with the default downregulation into sleep and prompting insomnia complaints.

**TABLE 1:** Cognitive factors identified across nine distinguishable theoretical models

Cognitive factors^a^		Theoretical models by author(s)								
		Borkovec	Morin	Perlis et al.	Lundh & Broman	Fichten et al.	Espie & Espie et al.	Harvey	Ong et al.	Rash et al.
Worry										
Dysfunctional cognitions	Worry over sleep loss									
	Rumination over consequences									
	Unrealistic expectations									
	[Amplification of insomnia consequences]									
	[Misattributions of the causes of insomnia]									
	Faulty beliefs about sleep promoting practices									
Cortical arousal	Enhanced sensory processing									
	Enhanced information processing									
	Enhanced long‐term memory function									
Sleep‐interfering processes	Negatively valenced thoughts									
	Worrying									
Sleep‐interpreting processes	Personal standards (e.g., perfectionism)									
	Attitudes, beliefs, and fears									
Negative cognitive activity										
Selective attention/attentional bias										
Explicit intention										
Sleep effort										
[Failure to de‐arouse (or cognitive de‐arousal)]										
Excessive negatively toned cognitive activity										
Arousal and distress										
Selective attention and monitoring										
Distorted perception of sleep/daytime performance										
Erroneous beliefs about sleep and the benefits of worry										
Safety behaviours										
Positive beliefs about worry										
Primary arousal	Expectations about sleep									
	Beliefs about daytime consequences of sleep loss									
	Increased mental activity in bed									
Secondary arousal [Metacognitive arousal]	Attentional bias									
	Rigidity in sleep‐related behaviour and beliefs									
	Attachment to sleep‐related needs and expectations									
	Absorption in solving the sleep problem									
Psychological flexibility	Acceptance									
	Cognitive defusion									
	Being present									
	Self‐as observer									
	Values									
	Committed action									

*Note*: “[]” = indicate rephrased terminology from original model publication. 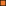 Cognitive factor(s) featured as a core component within the model, 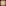 High similarity with constructs highlighted in other models, 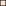 Low similarity with constructs highlighted in other models, 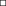 Cognitive factor(s) is not featured within the model.

^a^ Refer to Table 2 for definitions. Only cognitive components from theoretical models were included in this table. Similarity ratings to be interpreted within column, column by column.

A wide range of cognitive processes is described in the models identified. With subtle differences in definition and conceptualisation (i.e., a lack of common terminology), it is not an easy task to identify a lower‐level denominator to highlight shared commonalities. Although broadly speaking, most of the cognitive processes featured could be mapped onto general categories concerning thoughts, attention, perception, appraisal, and memory. Exceptions to that were components of psychological flexibility based on the acceptance and commitment therapy (ACT) framework, with emphasis not on the content and structure of the cognition but on the function and a person's relationship with the thoughts. We note that there are more validated instruments/tasks available for the assessments of the content and structure of cognition, than meta‐cognitions and processes proposed in ACT‐based models (Hiller et al., 2015). We also note that whilst the CBT models of insomnia tend to be formulated around the insomnia experience, ACT models of insomnia treatment tend to focus on the recovery process non‐specific to insomnia (Table 2).

**TABLE 2:** Conceptual and operational definitions of cognitive factors identified across theoretical models

Citation (year)	Cognitive factor(s)	Conceptual definition	Marker(s)/measure(s)^a^
Borkovec (1982)	Worry	Intrusive, uncontrollable thoughts and images that are attention‐grabbing in nature	None
Morin (1993)	Dysfunctional cognitions, including: Worry over sleep loss Rumination over consequences Unrealistic expectations about sleep requirements [Amplification of insomnia consequences] [Misattributions of the causes of insomnia] Faulty beliefs about sleep‐promoting practices	Learned affect‐laden, dysfunctional cognitions about sleep loss and its consequences that contribute to the perpetuation of insomnia	*Dysfunctional Beliefs and Attitudes About Sleep Scale* (*DBAS*) *Glasgow Content of Thoughts Inventory* Thought‐sampling methods during pre‐sleep period to assess the rate, content, and affective tone of cognitions
Perlis et al. (1997)	Cortical arousal, characterised by:	A form of conditioned arousal characterised by both “somatic” and “cognitive” arousal	*High frequency EEG activity* (i.e.*, in the Beta [14–32 Hz] and Gamma [>32 Hz] ranges*) *at or around sleep onset*
	Enhanced sensory processing	Increased level of responsiveness/vulnerability to perturbating environmental stimuli	
	Enhanced information processing	Increased perception of environmental stimuli	
	Enhanced long‐term memory function	Increased ability to encode and retrieve information	
Lundh and Broman (2000)	Sleep‐interfering processes, including: Negatively valenced thoughts Worrying	“Psychological processes that may have an influence on a person's sleep, independently of the person's interpretation of his or her sleep patterns or daytime consequences of poor sleep” (Lundh & Broman, 2000, p. 299) Examples include emotional conflicts, worries, and negative conditionings	None
	Sleep‐interpreting processes, including: Personal standards (e.g., perfectionism) Sleep‐related attitudes, beliefs, and fears	Psychological factors that “influence how the person interprets fluctuations in his or her sleep, sleep difficulties, and daytime consequences of poor sleep” (Lundh & Broman, 2000, p. 299) Examples include personal standards such as perfectionism, sleep‐related attitudes, beliefs, and fears	
Fichten et al. (2001)	Negative cognitive activity	“Negative, worrying, and anxious thoughts and self‐statements during periods of nocturnal wakefulness” (Fitchen et al., 2001, p. 666)	*Cognitive Content Questionnaire* (*CCQ*), a measure for assessing thought content and valence *“Self‐Statement Test: 60+”* to assess endorsement of valenced thoughts *Overall Thought Pleasantness Rating*
Espie (2002), Espie (2023), Espie et al. (2006)	Selective attention / Attentional bias	An attention bias towards sleep‐related stimuli, including attention focus on sleeplessness, sleep “deprivation” related worry, and longing for sleep	For “worry about sleep”: specific items from the Pre‐Sleep Arousal Scale (PSAS), *DBAS*, the *sleep disturbance questionnaire*, *the self‐statement test: 60C*, and *the Anxiety and Preoccupation about Sleep Questionnaire (APSQ)* Attention bias assessment via computerised experimental protocols including the Stroop task, dot probe, and flicker induced change blindness (ICB)
	Explicit intention	“Intentive actions designed to deliver sleep and to eliminate wakefulness” (Espie et al., 2006, p. 229)	Experimental comparison studies where people are instructed to fall asleep “as quick as possible” versus “whenever they would like”. Experimental manipulation studies where participants are instructed to suppress/not‐suppress thoughts, or to simply monitor thoughts
	Sleep effort	“A further development of intention” and characterised by a proactive behavioural state (Espie et al., 2006, p. 233). Examples include actively “trying to sleep” and increasing sleep opportunity	Individual items such as “*When I went to bed last night, I tried really hard to get to sleep” ranging from 0 “not at all” to 6 “very much*”, the *Glasgow Sleep Effort Scale* (*GSES*), and *specific items from the DBAS* and *SDQ scales*
	[Cognitive de‐arousal]	A natural/passive disengagement of information‐processing activities during the peri‐sleep period. This process is responsible for functional affect and cognitive regulation as evidenced by neutral affect and “few inaccurate expectations or worries about sleep or wakefulness” (Espie, 2002, p. 228). One of the four defensive properties of good sleep	Pre‐Sleep Arousal Scale (PSAS), *cognitive items of the Sleep Disturbance Questionnaire* (*SDQ*) Overall, measurement should *“be directed towards measuring multiple interacting inputs and outputs”* (Espie, 2023, p. 4) given the assumed interaction between the four subsystems (physiological, cognitive, affective, and behavioural)
Harvey (2002, 2005)	Excessive negatively toned cognitive activity	Unwanted intrusive and worrisome “excessive negatively toned cognitive activity about getting enough sleep and about the impact the sleep disturbance is having on health and/or daytime functioning.” (Harvey, 2002, p. 871) Such intrusive thoughts are described as closely related to worry and rumination	Pre‐sleep Intrusive Cognitions Inventory Cognitive items in the SDQ Impact of Event Scale Anxiety and Preoccupation About Sleep Questionnaire (APSQ) (Tang & Harvey, 2004b; Jansson‐Fröjmark et al., 2011; Norell‐Clarke et al., 2010) Insomnia Catastrophizing Scale (Jansson‐Fröjmark et al., 2012, 2020) Catastrophizing Interview (Harvey et al., 2003)
	Autonomic arousal and emotional distress	Arousal due to the activation of the sympathetic nervous system and “fight or flight” response, which results into an anxious state. Emotional distress is evidenced by states such as anxiety and dysphoria.	Nighttime measures: rectal temperature, vasoconstriction, skin conductance, body movement, metabolic rate
	Selective attention and monitoring of sleep‐related threat cues	Selective attention and automatic monitoring of internal (i.e., body sensations) and external/environmental sleep‐related threats	Assessment of pre‐sleep cognitive activity Sleep Associated Monitoring Index (Semler et al., 2004) Experimental manipulation of monitoring by asking people to “monitor the clock” versus “not monitor the clock” (Tang et al., 2007)
	Distorted perception of sleep/daytime performance	Distorted perception of the amount of sleep obtained and associated daytime deficits	Assessment of pre‐sleep cognitive activity Sleep amount distortion: multiple sleep latency testing (MSLT), pupillometry, and neuropsychological testing
	Erroneous beliefs about sleep and the benefits of worry	Overestimation of “the extent of the perceived deficit in sleep and daytime performance” (Harvey, 2002, p. 872)	As described by Morin (1993) – i.e., DBAS
	Safety behaviours	Counterproductive behaviours used to cope with excessive cognitive activity “including thought control, imagery control, emotional inhibition, and difficulty problem solving” (Harvey, 2002, p. 869)	Sleep Related Behaviours Questionnaire (Ree et al., 2004) Thought Control Questionnaire – Insomnia Revised (Ree et al., 2005) Assessment of pre‐sleep cognitive activity
	Positive beliefs about worry	A type of metacognition (i.e., self‐awareness about one's thoughts and thought processing) characterised by “positive beliefs about the benefits of worrying in bed”. An example of such positive belief would be thinking that worrying in bed “helps to sort out/put things in order in my mind” (Harvey, 2005, p. 102)	Utility of Pre‐sleep Worry Questionnaire (UPWQ) (Harvey, 2003)
			Overall, both night and daytime processes are assumed to equally contribute in the maintenance of insomnia. Thus, author suggests studies should include outcome measures of daytime functioning
Ong et al. (2012)	Primary arousal, including: Expectations about sleep Beliefs about daytime consequences of sleep loss Increased mental activity in bed	“Primary arousal consists of the cognitive activity directly related to the inability to sleep. This includes the thoughts that interfere with sleep and the beliefs about daytime consequences of poor or insufficient sleep” (Ong et al., 2012, p. 653)	None
	Metacognitive arousal (secondary arousal), including: Rigidity Attentional bias Attachment Absorption	As opposed to primary arousal (i.e., arousal caused by cognitions), secondary arousal relates to metacognitions – i.e., “how one relates to thoughts about sleep” (Ong et al., 2012, p. 653) and it is proposed as a perpetuating mechanism for insomnia	Metacognitive Questionnaire for insomnia (MCQ‐I)
		Rigidity in sleep‐related behaviour and beliefs	None
		Excessive “attention and emotional bias towards sleep‐seeking or sleep aversive thoughts and behaviours” (Ong et al., 2012, p. 655)	None
		“Attachment to sleep‐related needs and expectations” (Ong et al., 2012, p. 655)	None
		Over absorption in solving the sleep problem	None
Rash et al. (2019)	Psychological flexibility, consisting of:	The term “refers to (1) conscious and open contact with thoughts and feelings, (2) an awareness of the available responses in any situation, and (3) changing or persisting in behaviour when doing so serves one's goals and values” (Rash et al., 2019, p. 331)	None
	Acceptance	Openness to undesired experiences without struggle or avoidance	
	Cognitive defusion	Being able to notice thoughts by what they are and not getting “caught up” in their meaning	
	Being present	“Open, nonjudgemental contact with the present” (Rash et al., 2019, p. 331)	
	Self‐as‐observer	Perception of oneself beyond thoughts and feelings	
	Values	“Understanding chosen qualities of purposive action” (Rash et al., 2019, p. 331)	
	Committed action	“Development of larger patterns of effective actions linked to chosen values” (Rash et al., 2019, p. 331)	

*Note*: “[]” = indicate rephrased terminology from original model publication.

Abbreviations: APSQ, Anxiety and Preoccupation about Sleep Questionnaire; EEG, Electroencephalography; DBAS, Dysfunctional Beliefs and Attitudes About Sleep Scale; PSAS, Pre‐Sleep Arousal Scale; SDQ, Sleep Disturbance Questionnaire.

^a^ Marker(s)/measure(s) suggested by the author are *italicised*; all other possible measures are indicated in the manuscript as used by other authors.

Despite the cognitive emphasis of some of the identified models, the cognitive processes were seldom proposed in isolation from the physiological experience of insomnia. On the contrary, the majority of these models acknowledged insomnia as a multi‐level experience and made specific links to the (hyper)arousal concept to make plain how cognitions weave the psychological experience of insomnia with the physiological phenomenon of sleeplessness (Riemann et al., 2010). For example, in the Perlis et al. (1997) model, cortical arousal as indicated by high frequency EEG activity were thought to be reflecting enhanced sensory processing, information processing, and memory function. In the models by Borkovec (1982), Morin (1993), Lundh and Broman (2000), Fichten et al. (2001), Harvey (2002), and Ong et al. (2012), worry and increased negatively toned cognitive activity were thought to lead to arousal that interferes with sleep onset mechanisms. And finally in Espie's PIM model, attention and intention to sleep and sleep effort were thought to be cognitive factors that prevent natural de‐arousal associated with normal sleep onset. These cognitive factors might also have a disruptive impact on the default settings of the sleep homeostat and circadian timing system.

## 4 DISCUSSION

The current review revealed a vibrant scene over the past four decades, with nine distinguishable models of insomnia featuring at least one cognitive factor. This affirms the traction of cognition as an important factor explaining the development, maintenance, and/or remission of insomnia. There was a surprising amount of similarity between models. Among the 20–39 cognitive factors and processes identified, our analysis suggested that “worry”, “rumination”, “enhanced information processing”, “negatively valenced thoughts”, “excessive negatively toned cognitive activity”, “autonomic arousal/emotional distress”, and “primary arousal” are the most cross‐cutting cognitive factors among models.

Most of the cognitive processes shared across models continue to receive widespread research attention. In particular, cognitive arousal appearing as worry and/or rumination, whereby findings have suggested that – whilst both are repetitive thought processes – they are separate constructs with different contents (e.g., verbal thoughts vs. images), focus (e.g., what if vs. why), perspective (e.g., future vs. past), and possibly effects on emotion regulation and insomnia symptom presentation (Carney et al., 2010b, 2013; Galbiati et al., 2018). Timing and state of appearance also seem to make a difference with night time sleep‐related worry found to be have a stronger association with insomnia symptoms, compared with daytime worry and one's general tendency to ruminate (Lancee et al., 2017). This is consistent with other studies emphasising the nocturnal timing of cognitive arousal (Kalmbach et al., 2020) and that sleep‐related cognitive arousal was more closely associated with measures of sleep onset and maintenance problems than general cognitive arousal without a sleep focus (Spiegelhalder et al., 2012). It is also consistent with a 2020 meta‐analysis of 15 randomised controlled trials (RCTs) that has found a significant moderate to large effect of CBT‐I in reducing worry (Ballesio et al., 2021), with effect on sleep‐related worry [as measured with the Anxiety and Preoccupation About Sleep Questionnaire, and Dysfunctional Beliefs About Sleep questionnaire (Morin et al., 2007; Tang & Harvey, 2004a)] being larger than that on general worry [as measured with the Penn State Worry Questionnaire, and Worry Domains Questionnaire (Jansson‐Fröjmark & Linton, 2007; Meyer et al., 1990)]. The effect of CBT‐I on rumination reduction was non‐significant and small, although the null effect may be explained by the small number of RCTs including rumination as an outcome measure and that there was high heterogeneity between studies.

Relatedly, another widely investigated cognitive construct is dysfunctional beliefs and attitudes about sleep. Over the years, the DBAS scale has received validations in its original 30‐item version (Chung et al., 2016; Espie et al., 2000; Morin, 1993) as well as in shorter versions of 16 items (DBAS‐16) (Carney et al., 2010a; Morin et al., 2007) and of 10 items (DBAS‐10) (Edinger & Wohlgemuth, 2001; Espie et al., 2000). With empirical research, we have come to understand that these unhelpful beliefs occur not only in adults but also in children (Gregory et al., 2009; Schneider et al., 2019). It has been demonstrated in prospective studies that DBAS – alongside other cognitive‐behaviour factors – is associated with the maintenance of insomnia symptoms (Jansson & Linton, 2007; Norell‐Clarke et al., 2014). Thankfully, DBAS is responsive to CBT‐I. A 2020 meta‐analysis of 16 RCTs found a large effect of CBT‐I in reducing dysfunctional beliefs about sleep at post‐treatment, with effect maintained at follow‐up from 3 to 18 months (Thakral et al., 2020). It is important to note that reduction in DBAS is a predictor of both self‐reported and polysomnography‐measured treatment gains (Morin et al., 2002). Further, multiple analyses of temporally structured data from RCTs or prospective cohort studies have shown that changes in DBAS is a significant mediator of improvement in insomnia symptoms following CBT‐I (Parsons et al., 2021; Sunnhed et al., 2022). Together, these findings build a strong case for a causal role of unhelpful beliefs (as a cognitive factor) in the maintenance and remission of insomnia. The clinical significance of unhelpful beliefs however does not stop here, as in recent years, the DBAS has also been found to predict and to help understand insomnia comorbid with cancer (Tremblay et al., 2009), schizophrenia (Chiu et al., 2015), chronic pain (Afolalu et al., 2018), myocardial infarction (Da Costa et al., 2017), as well as the link from insomnia to suicide (McCall & Black, 2013).

Interest has grown in the role of metacognitive processes with the advent of the third‐wave therapies and the emergence of process‐based insomnia models (Ong et al., 2012; Rash et al., 2019). There have been studies showing an association between psychological flexibility and sleep quality in people with chronic pain, whereby all components of psychological flexibility together account for up to 19% of variance in insomnia symptoms (McCracken et al., 2011). Of the six components of psychological flexibility, mindfulness is the most studied in terms of its role in insomnia and emotion regulation. It has been proposed that mindfulness‐based interventions are a feasible adjunct or alternative to CBT‐I (Ong & Kalmbach, 2023). This is not out of the blue, as a 2019 meta‐analysis of 19 trials has found moderate evidence that mindfulness‐based interventions improved sleep quality with durability up to 5–12 months compared with non‐specific active controls. However, we note that no significant effect on sleep quality was found when compared with evidence‐based active controls. Relatedly, a 2016 meta‐analysis of six earlier RCTs found the mediation by mindfulness to be tentative and possibly limited to self‐reported total wake time and sleep quality but not to other sleep parameters (e.g., SOL, WASO, TST, SE) or outcome measures (ISI, PSQI, DBAS) (Gong et al., 2016). Further research would certainly help to ascertain the effect of mindfulness‐based interventions on sleep and whether enhanced mindfulness is a critical mediator of therapeutic change. It would also be interesting to evaluate whether matching treatment approaches with conceptual targets (CBT‐I targeting dysfunctional beliefs and attitudes about sleep vs. Mindfulness targeting unhelpful metacognitions) would help move us towards precision medicine for insomnia. We should stress though that this area of research is rapidly evolving, with growing interest in using ACT‐based therapies for insomnia. ACT has great emphasis on the components of psychological flexibility other than mindfulness, such as acceptance, committed action, cognitive defusion, self as context, and values (Paulos‐Guarnieri et al., 2022). There is also novel work investigating the bidirectional link between self‐compassion and sleep (Rakhimov et al., 2022a, 2022b) and the role of wider metacognition in mediating/regulating of arousal and sleep quality (Palagini et al., 2017; Zamani et al., 2022).

It is important to note that the conceptualisations identified in the current systematic review are primarily devised for the understanding of insomnia in adults. The extent to which the cognitive elements can be applied to understanding insomnia in children and adolescents is yet to be determined. That said, cognitive arousal (in the form of fear and worry) has been implicated in the presentation of insomnia in children as young as 8–10 (Gregory et al., 2008) with elevated level of cortical arousal (in the form of beta EEG power) in adolescents with insomnia during sleep onset and different NREM sleep stages (Fernandez‐Mendoza et al., 2016). Further, there is evidence from a 2018 meta‐analysis of six RCTs that cognitive and behavioural sleep interventions have a therapeutic effect on sleep in school‐aged children and adolescents (Åslund et al., 2018). Whilst components of these sleep interventions varied between RCTs, six key components were identified and these included typical components of CBT‐I, namely sleep education, sleep hygiene, sleep restriction, stimulus control, cognitive therapy, relaxation/mindfulness. These interventions target cognitions to the extent that all of the six RCTs included a sleep education component, three of them included a cognitive therapy component, and 3 of them included a relaxation/mindfulness component.

### 4.1 Limitations

The current systematic review represents an effort to comprehensively search and identify relevant human models of insomnia that feature cognitions in the explanatory framework. Whilst the number of theoretical articles identified is larger than what was reported in earlier narrative reviews (Espie, 2007; Talbot & Harvey, 2016), our search was limited to only two electronic databases deemed to be most relevant to the publication of psychological theories of insomnia (Medline and PsycInfo) and did not actively include grey literature and MSc/PhD theses. Although our search was topped up with consultations with experts in the field, the current systematic review may have missed out novel or unconventional theories that await further development and testing.

We should also note that our search did not go beyond the English language restriction. There was limited cultural diversity in the articles identified, and hence in the understanding of cognitive factors involved in the development and maintenance of insomnia across cultures. Large scale epidemiological studies have revealed that insomnia is also highly prevalent in non‐English speaking countries (Ohayon, 2002; Soldatos et al., 2005). It would be interesting from an anthropological perspective to investigate the extent to which language and cultures shape our cognitive experience of and response to insomnia (Knutson, 2013).

Due to the theoretical nature of the information being synthesised in the current systematic review, we are aware of the potential conscious and unconscious biases associated with our own training backgrounds, theoretical orientations, and familiarity of the different models. To minimise these, we adopted a systematic and structured approach to our data extraction and followed the PRISMA guidelines in our conduct and reporting of our findings. We also embraced recommendations for qualitative research to declare an interpretative element in our findings and be transparent our training backgrounds, theoretical orientations and roles in the process (e.g., CORE‐Q) (Tong et al., 2007).

## 5 CONCLUSION

Within the bounds of the above limitations, our systematic review has provided a timeline of cognitive factors and processes proposed to explain the development, maintenance and/or remission of insomnia. The number and variety of models suggests a “let a hundred flowers bloom” scenario, and given the growing empirical evidence supporting a causal role of cognition in insomnia, we believe the field has moved past the idea that cognitive arousal is a mere epiphenomenon of sleeplessness. The recent expansion to include metacognitions and generic ACT‐based processes for the consideration of insomnia may serve well in offering new treatment avenues, particularly for insomnia occurring in a multi‐comorbidity context. However, progress on this front would depend on researchers’ and clinicians’ theoretical uptake and the availability of validated instruments to measure the featured cognitive processes. In fact, the need to unify terminologies, definitions and assessment methods is shared by cognitive constructs featured in CBT models as well. We need better tools to assess cognitive factors and processes, and the development of appropriate tools should be aided by better understanding of the key components or latent dimensions of the identified cognitive factors and processes. We recommend future research to apply empirical approaches to examine these and to profile the neurophysiological correlates of each key component and latent dimension. This we believe would provide a foundation for identifying core cognitive outcomes for future clinical trials and enhancing comparability between studies, serving as a stepping stone towards advancing the field. Finally, all of the models identified in the current systematic review were developed based on human data collected in experimental and clinical contexts. With the advent of AI technology and the widespread use of digital CBT‐I and other health apps, alternative approaches to insomnia modelling are on the horizon. In the near future, we may witness a surge of computational cognitive models of insomnia that utilise big data simulations and machine learning algorithms for theory generation, development, and evaluation (Haslbeck et al., 2022; Tai et al., 2019).

## AUTHOR CONTRIBUTIONS

**Nicole K. Y. Tang:** Conceptualization; data curation; funding acquisition; investigation; methodology; project administration; resources; supervision; visualization; writing – original draft; writing – review and editing. **Bruno Saconi:** Conceptualization; data curation; formal analysis; methodology; project administration; software; visualization; writing – original draft; writing – review and editing. **Markus Jansson‐Fröjmark:** Conceptualization; validation; writing – review and editing. **Jason Ong:** Conceptualization; validation; writing – review and editing. **Colleen Carney:** Conceptualization; validation; writing – review and editing.

## FUNDING INFORMATION

NT is supported by Medical Research Council (MRC) grant number: MR/S026185/1. Her research on insomnia and chronic pain was funded by the National Institute for Health Research under its Research for Patient Benefit (RfPB) Programme (Grant Reference Number PB‐PG‐0213‐30121). The views expressed are those of the author(s) and not necessarily those of the NHS, the NIHR or the Department of Health and Social Care.

## CONFLICT OF INTEREST STATEMENT

The authors have no conflict of interest to disclose. Jason Ong received salary from Nox Health.

## Supporting information


**Appendix S1:** Supplementary Information

## Data Availability

N/A.
